# TPX2 promotes ovarian tumorigenesis by interacting with Lamin A/C and affecting its stability

**DOI:** 10.1002/cam4.5683

**Published:** 2023-02-15

**Authors:** Xin Meng, Jiazhen Cao, Hui Zheng, Xiaolu Ma, Yanchun Wang, Ying Tong, Suhong Xie, Renquan Lu, Lin Guo

**Affiliations:** ^1^ Department of Clinical Laboratory Fudan University Shanghai Cancer Center Shanghai China; ^2^ Department of Oncology, Shanghai Medical College Fudan University Shanghai China

**Keywords:** Lamin A/C, ovarian cancer, TPX2

## Abstract

**Objective:**

Ovarian cancer (OC) is one of the fatal gynecologic malignancies. However, there are no effective prognostic or therapeutic indicators for OC. Herein, we aim to reveal the potential function of targeting protein for Xklp2 (TPX2) in OC progression.

**Methods:**

Immunohistochemical and bioinformatic analyses were used to evaluate the level of TPX2 in OC samples. Effects of TPX2 on cell proliferation, cell apoptosis and ROS production were evaluated in vivo and in vitro. Mass spectrometry, Co‐IP and immunofluorescence assays were performed to identify and verify protein‐protein interactions.

**Results:**

Our data showed that pathological overexpression (OE) of the TPX2 in OC could manifest a poor prognosis. Functional studies demonstrated that TPX2 silencing led to the suppression of cell proliferation in vitro and in vivo through an increase in reactive oxygen species (ROS) level and apoptosis, while TPX2 OE exhibited the opposite effect. Furthermore, by mass spectrometric analysis, we identified a novel interacting partner, Lamin A/C, for TPX2. Mechanistically, TPX2 regulated Lamin A/C's stability by modulating the level of phospho‐Lamin A/C (Ser 22).

**Conclusion:**

Our findings thus suggest that TPX2 may be a promising therapeutic target for OC.

## Graphical abstract

TPX2 can interact with Lamin A/C and modulate its stability by regulating the level of p‐Lamin A/C (Ser22) and degradation of Lamin A/C in OC cells, targeting cell apoptosis.

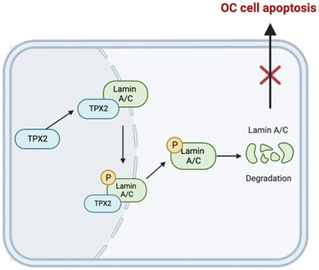

## 1 INTRODUCTION

Ovarian cancer (OC) accounts for the fifth highest rate of cancer‐related death among women worldwide. American Cancer Society data indicate an estimation of 19,880 cases to be diagnosed with OC in 2022, with 12,810 estimated deaths.^1^ Among all OC subtypes, epithelial OC alone accounts for 90% of cases. Because of the difficulty in the early detection of OC, most patients progress to the advanced stage by the time they are diagnosed. So far, combination chemotherapy (platinum–taxane) and cytoreductive surgery have remained the conventional treatment options for OC.^2^ However, most patients ultimately acquire chemoresistance within a few years, despite their initial responses to the therapy.^3^ Moreover, there are no approved immune therapies for OC,^4^ warranting an urgent need for developing precision therapies for OC.

The targeting protein for Xklp2 (TPX2), a microtubule‐associated factor, functions in the mitotic spindle assembly process during cell division.^5^ Abnormal TPX2 expression can lead to spindle abnormalities and aberrant centrosome amplification. Additionally, TPX2 OE has been detected in several cancers, including breast cancer, liver cancer, gastric cancer, pancreatic cancer, and bladder cancer, strongly linking to a poor cancer prognosis.^6^, ^7^, ^8^, ^9^, ^10^ Although TPX2 is observed to be overexpressed in OC,^11^ the precise mechanisms underlying its functions require further investigation.

Here, we aimed to delineate  the oncogenic role of TPX2 in OC tumorigenesis and its possible utilization as a potential biomarker through a series of functional and bioinformatic analyses. We confirmed that TPX2 was an etiopathological factor in OC onset and associated with poor prognosis. TPX2 accelerated tumor growth via suppressing apoptosis and ROS production in OC cells by modulating Lamin A/C's stability.

## 2 MATERIALS AND METHODS

### 2.1 Cell culture and patient samples

A2780, SKOV3, and HEK‐293T cell lines were maintained either in RPMI‐1640 or DMEM medium, added with 1% penicillin–streptomycin (P/S), and 10% of fetal bovine serum. Cells were cultured at 37°C in a humidified incubator with 5% CO_2_. A total of 108 primary OC samples and normal ovarian tissues were collected at the Fudan University Shanghai Cancer Center. Informed consent was signed by all eligible participants.

### 2.2 Immunohistochemical (IHC) analysis

IHC analysis for TPX2 and Lamin A/C was performed on the tissue microarray using anti‐TPX2 (CST, #12245), and anti‐Lamin A/C (CST, #13448) antibodies, followed by a secondary antibody (Long Island Antibody, #D‐3004) staining. The expressions of TPX2 and Lamin A/C in OC samples were measured based on an IHC scoring system (intensity: 0 = negative, 1 = weak, 2 = moderate, and 3 = strong; extensity: 0 = denotes <5% of positive cells; 1 = 5%–25%, 2 = 26%–50%, 3 = 51%–75%, and 4 = denotes >75%). The final score was a product of the intensity score and the extensity score.

### 2.3 Lentiviruses and siRNA transfection

TPX2‐OE and vector plasmid were purchased from Genecham (China). Lentiviral was generated from HEK293T cells, following the manufacturer's protocol. Then, A2780 and SKOV3 cells were transduced with lentiviral particles, along with 8 μg/mL of polybrene for 24 h. Cells were then selected in the puromycin‐containing medium for 3 days. TPX2‐knockdown (KD) cells were also established by lentivirus infection. The lentivirus was from Genecham (China). Antisense RNA against Lamin A/C (siLMNA) and negative control siRNA (Sangon Biotech) were transfected in OC lines using lipofectamine‐2000 (Invitrogen) reagent. Table S1 enlists the shRNA and siRNA sequences used in this study.

### 2.4 Western blotting (WB) analysis

RIPA lysis buffer (Beyotime, #P0013C) with 1X protease inhibitors was used for cell lysate preparation. Protein concentrations were estimated by the BCA method (YEASEN, #20201ES76) and resolved by running on SDS‐PAGE. Proteins were subsequently transferred onto PVDF membranes for WB analysis. After blocking in 5% of non‐fat milk (Sangon Biotech, #A600669), blots were probed with anti‐GAPDH (Proteintech, #10494‐1‐AP, 1:3000), anti‐TPX2 (CST, #12245, 1:500), anti‐Bcl2 (CST, #15071, 1:1000), anti‐Bax (CST, #5023, 1:1000), anti‐Lamin A/C (CST, #4777, 1:1000), and anti‐phospho‐Lamin A/C (CST, #13448, 1:1000) antibodies overnight at 4°C, followed by incubation with HRP‐conjugated Goat anti‐Rabbit IgG (Proteintech, #SA00001‐2, 1:3000), or Goat anti‐Mouse IgG (Proteintech, #SA00001‐1, 1:3000) secondary antibody for 2 h at room temperature (RT).

### 2.5 Colony‐forming assay

A total of 1000 OC cells/well were plated in six‐well plates and cultured for about 10–14 days, followed by being fixed in 4% paraformaldehyde (PFA) and stained with crystal violet. The number of visible colonies was counted manually.

### 2.6 EdU assay

A2780 and SKOV3 cells were seeded at 2 × 10^5^ cells/well in six‐well plates and treated with 50 μM of EdU solution for 2 h at 37°C, followed by 4% PFA fixation for 30 min and reaction quenching by 2 mg/mL of glycine solution. Cells were permeabilized with a buffer containing 0.5% of Triton X‐100 for 10 min at RT. Cells were then stained with 1× EdU solution, washed with the permeabilization buffer, and analyzed by flow cytometry.

### 2.7 Apoptosis assay

A2780 and SKOV3 cells were plated at 3 × 10^5^ cells/well in six‐well plates, incubated with 7‐AAD and PE Annexin V (BD Biosciences, #559763), and subjected to flow cytometry for analysis.

### 2.8 ROS assay

A2780 and SKOV3 cells were seeded at 3 × 10^5^ cells/well in six‐well plates and treated with 5 μM of DCFH‐DA for 30 min at 37 °C, and ROS levels were quantified by flow cytometry.

### 2.9 Co‐immunoprecipitation (Co‐IP)

Cells were lysed in IP lysis buffer (Beyotime, #P0013J) with protease inhibitors. Lysates were incubated with either target antibodies or normal IgG overnight at 4°C, followed by incubating with protein‐A/G agarose beads (SCBT, #sc‐2003) at 4°C for 2 h. After washing with lysis buffer, beads were incubated with SDS loading buffer at the boiling condition for 10 min for WB.

### 2.10 Mass spectrometry (MS)

To characterize TPX2 interactomes, anti‐TPX2 immunoprecipitated proteins were subjected to MS analysis at Luming Biotech (China). Electrophoresis followed by Coomassie blue dye staining confirmed the presence of Lamin A/C in TPX2 IP eluates.

### 2.11 Immunofluorescence (IF) staining

A2780 and SKOV3 cells were fixed with 4% PFA, permeabilized using 0.5% of Triton X‐100, blocked with 3% of BSA, and labeled with anti‐TPX2 (CST, #12245, 1:100), and anti‐Lamin A/C (CST, #4777, 1:50). Next, slides were washed thrice with PBS and labeled with fluorescent‐conjugated secondary antibodies for 2 h at RT in the dark, and nuclei were counter‐stained with DAPI. Images were captured and analyzed on a confocal microscope Lecia STELLARIS 5.

### 2.12 Animal experiments

The protocol was approved by the Animal Experiments Committee of Fudan University Shanghai Cancer Center. A2780‐derived xenograft model was created in BALB/c nude female mice (4‐ to 6‐week‐old) by subcutaneous injection. Tumor sizes were measured and analyzed.

### 2.13 Statistical analysis

GraphPad Prism 8 was utilized for all statistical analyses, and data were presented as mean ± SD. Between the groups, analyses were carried out either by Student's *t*‐test, ANOVA, or Wilcoxon test. The results with a *P*‐value of < 0.05 were considered significant.

## 3 RESULTS

### 3.1 TPX2 is overexpressed in OC and predicts a poor prognosis

**FIGURE 1:**
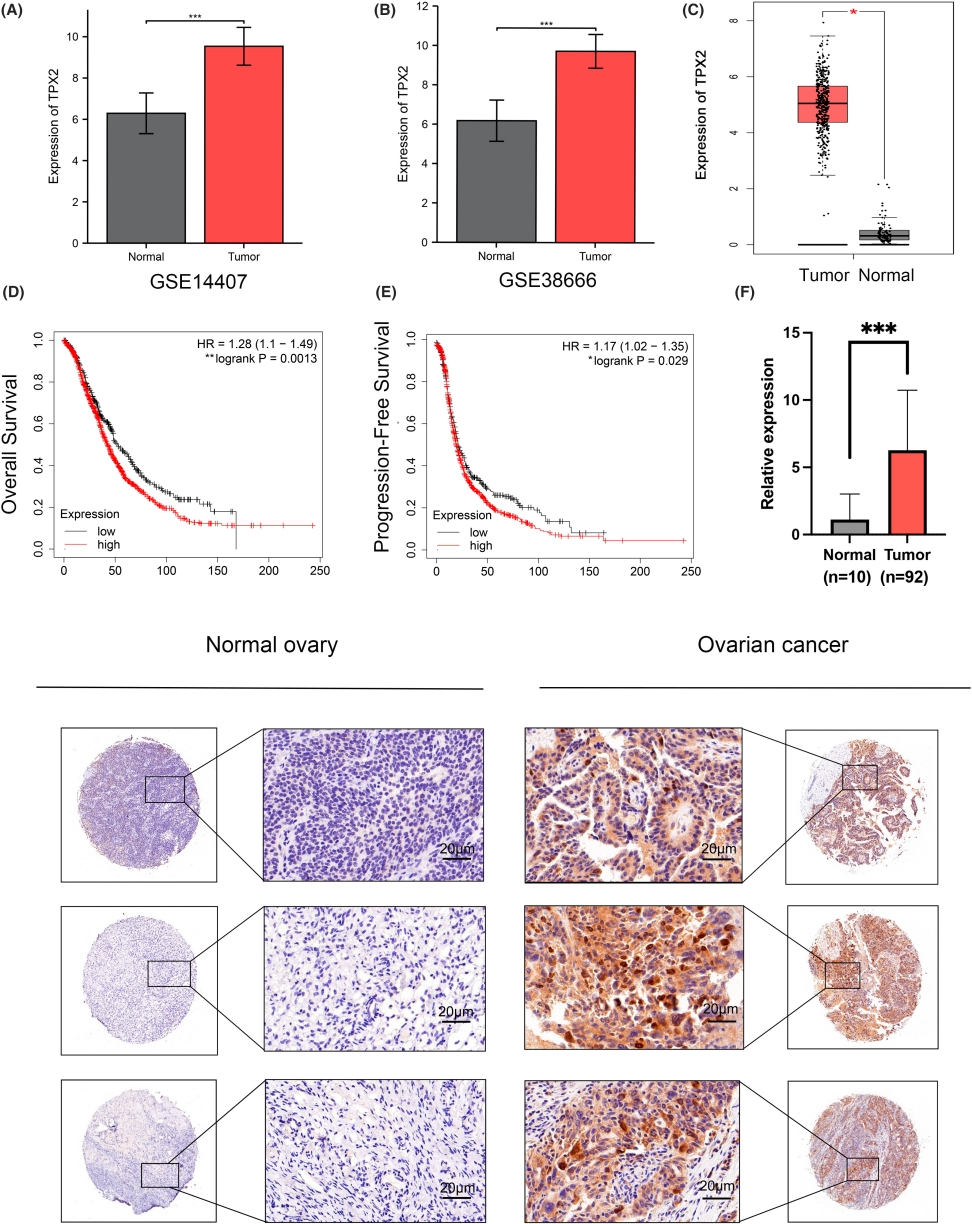
The targeting protein for Xklp2 (TPX2) is overexpressed in ovarian cancer (OC) and predicts a poor prognosis. (A–B) Protein levels of TPX2 in OC tissues and normal tissues from the GEO database. (C) Comparison of TPX2 levels between OC and normal tissues in TCGA database. The figure was retrieved from the GEPIA database. (D–E) Overall survival (OS) and progression‐free survival (PFS) of OC patients with high or low levels of TPX2. The images were from the Kaplan–Meier database. (F) Representative immunohistochemical (IHC) staining images and IHC scores of TPX2 in OC and normal tissues. *p<0.05, **p<0.01, ***p<0.001.

To explore the mechanistic role of TPX2 in OC pathogenesis, we analyzed TPX2 expression profiles in both GEO and TCGA databases, which revealed its high expression in OC cells compared to that in normal healthy epithelial cells **(**Figure 1A–C **)**. To further support this finding, we assessed TPX2 levels in a cohort of primary OC tissues by IHC analysis **(**Figure 1F **)**, indicating elevated TPX2 signals of OC cells than those in the normal tissues. Furthermore, Kaplan–Meier survival curves showed that patients having higher levels of TPX2 expressions exhibited shorter overall survival (OS) and progression‐free survival (PFS) compared to their low‐expression counterparts **(**Figure 1D,E **)**. Thus, our findings demonstrate that TPX2 was overexpressed in OC and associated with poor prognosis in OC patients.

### 3.2 TPX2 accelerates tumor growth both in vivo and in vitro

**FIGURE 2:**
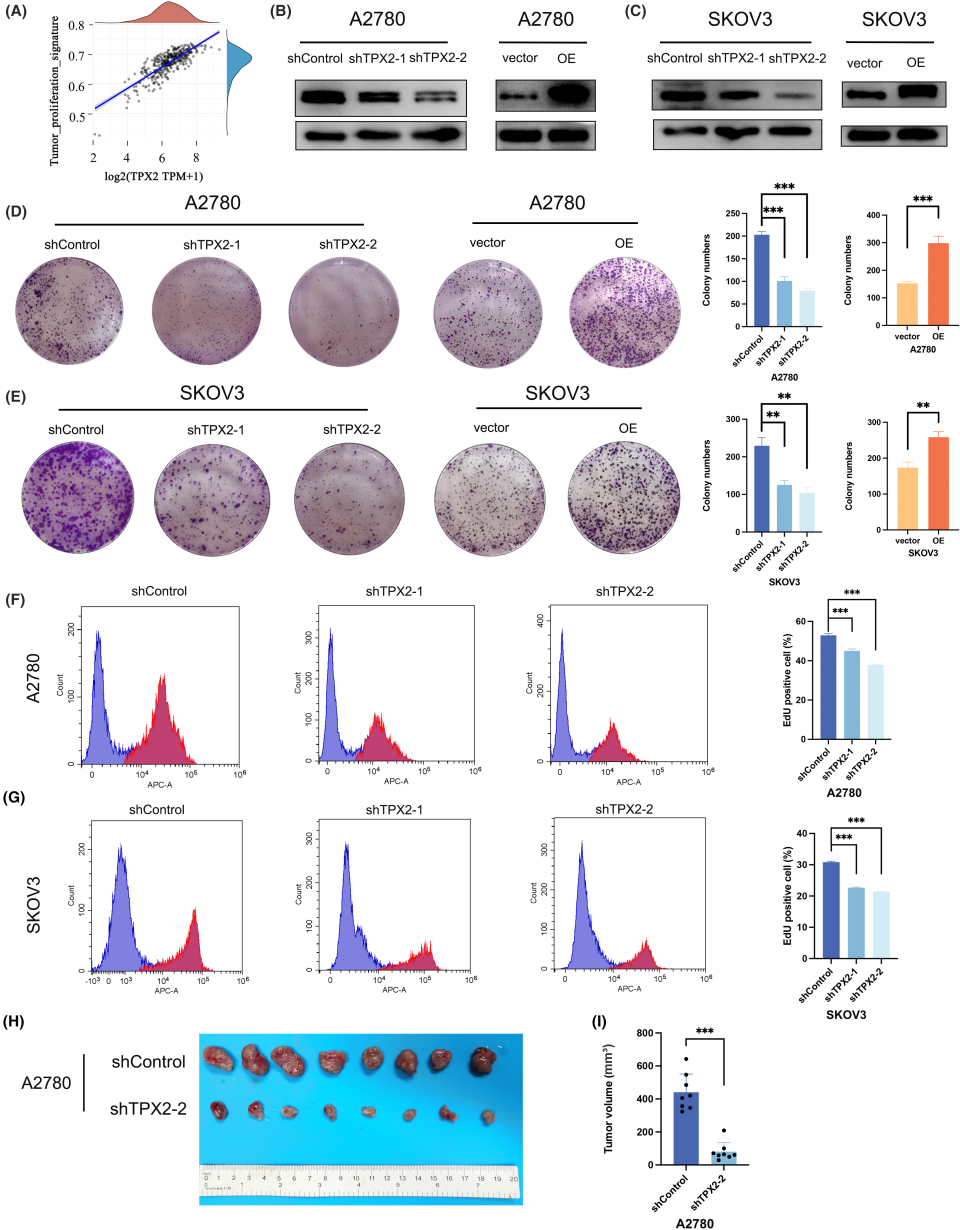
TPX2 accelerates tumor growth in vivo and in vitro. (A) Correlation of TPX2 level with tumor proliferation signature. (B–C) Knockdown (KD) and overexpression (OE) of TPX2 in A2780 and SKOV3 cells. (D–E) Colony formation assays were performed in A2780 and SKOV3 cells with TPX2‐KD or TPX2‐OE. (F–G) Cell proliferation ability was evaluated by EdU assay in A2780 and SKOV3 cells with TPX2‐KD or TPX2‐OE. (H) Images of TPX2 KD or the control xenografts formed from A2780 cells. (I) Volume of xenograft tumors. *p<0.05, **p<0.01, ***p<0.001.

TPX2 levels were positively connected with tumor proliferation signature **(**Figure 2A **)**. To further explore the biological functions of TPX2 in carcinogenesis, TPX2 was either knocked down or overexpressed in A2780 and SKOV3 cells via lentiviral transduction, and protein levels were analyzed by WB **(**Figure 2B,C **)**. TPX2 knockdown (KD) reduced colony formation, whereas TPX2 OE exhibited the reverse effect **(**Figure 2D,E **)**. The EdU assay showed that TPX2 depletion impaired the DNA synthesis ability, which was consistent with TPX2's role in promoting the proliferation of OC cells **(**Figure 2F,G **)**. Next, we examined whether TPX2 also could promote ovarian tumorigenesis in vivo by implanting A2780 cells into female nude mice. Tumor sizes were less in the TPX2 KD group compared to those in the control group **(**Figure 2H,I **)**, suggesting that TPX2 can accelerate tumorigenesis of OC cells.

### 3.3 TPX2 suppresses apoptosis and reduces ROS levels in OC cells

**FIGURE 3:**
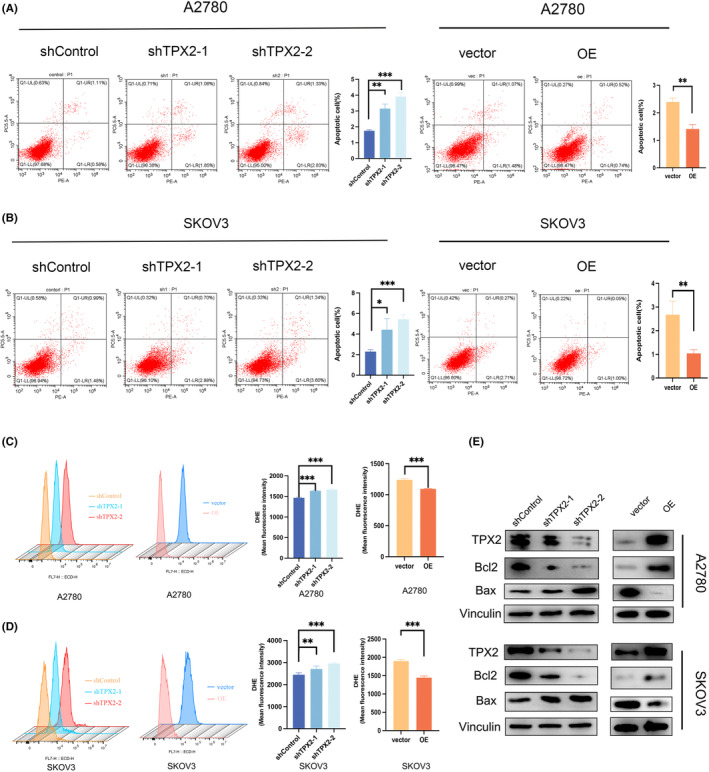
TPX2 suppresses apoptosis and reduces reactive oxygen species (ROS) levels in OC cells. (A–B) Cell apoptosis was measured by flow cytometry in A2780 and SKOV3 cells with TPX2‐KD or OE. (C–D) ROS levels were evaluated by flow cytometry in A2780 and SKOV3 cells with TPX2‐KD or OE. (E) Proteins levels of Bcl2 and Bax were detected by western blot.  **p<0.01, ***p<0.001.

Flow cytometry analysis of apoptosis indicated that TPX2 KD significantly increased the apoptosis rate in OC cells, while TPX2 OE had a reverse effect **(**Figure 3A,B **)**. Consistent with these results, there was an increase in the Bax level, but the Bcl‐2 level was decreased in the TPX2 KD group **(**Figure 3E **)**. Besides, we measured ROS levels in TPX2 OE and KD cells using the DCFDA assay. TPX2 KD elevated the ROS level, while its OE inhibited ROS production **(**Figure 3C,D **)**. These results suggest that TPX2 can suppress apoptosis and reduce ROS production in OC cells.

### 3.4 TPX2 interacts with Lamin A/C and affects its stability

**FIGURE 4:**
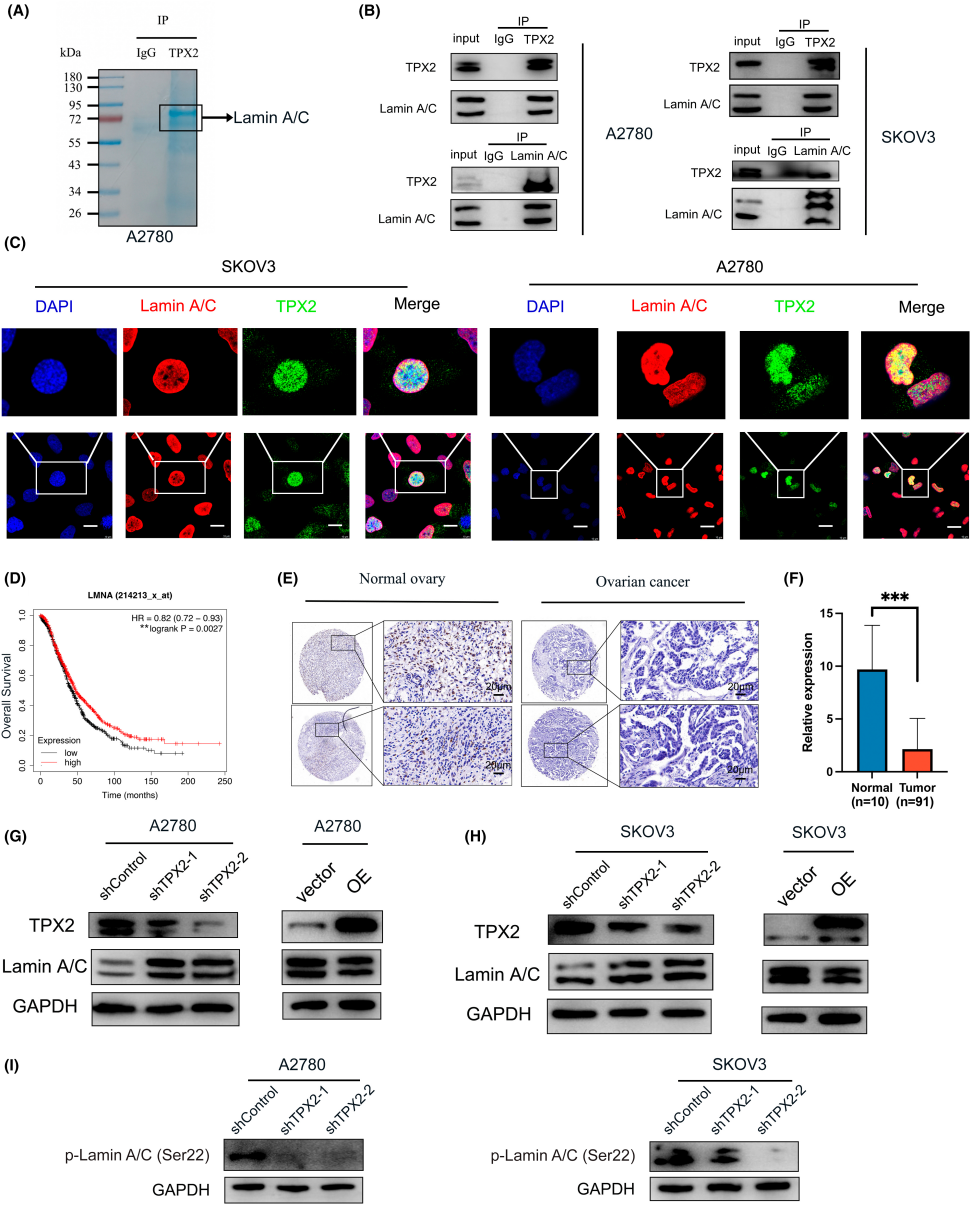
TPX2 interacts with Lamin A/C and affects its stability. (A) Representative Coomassie blue staining images of anti‐TPX2 or normal IgG immunoprecipitated proteins from A2780 cells. (B) Co‐immunoprecipitation analysis was performed to detect the interaction of TPX2 with Lamin A/C. (C) Immunofluorescence (IF) assay was used to confirm TPX2's colocalization with Lamin A/C in A2780 and SKOV3 cells. Scale bars: 10 μm. (D) OS of OC patients with high or low levels of LMNA. The images were from the Kaplan–Meier database. (E–F) Representative IHC staining images and IHC scores of Lamin A/C in OC and normal tissues. (G–H) Lamin A/C levels were detected in A2780 and SKOV3 cells with TPX2‐KD or OE. (I) Phospho‐Lamin A/C levels were detected in OC cells with TPX2‐KD.

To further elucidate how TPX2 promotes OC pathogenesis, anti‐TPX2 immunoprecipitated eluates from A2780 cells were subjected to the MS analysis for identification of its interacting partners. Notably, Lamin A/C was found to be the predominant one. Immunoprecipitated proteins were stained with Coomassie blue dye to detect bands of 72–95 kDa size in the experimental but not in the IgG control sample, which was speculated as a Lamin A/C protein band **(**Figure 4A **)**. We also confirmed the interaction between TPX2 with Lamin A/C by Co‐IP and IF analysis **(**Figure 4B,C **)**. Lamin A/C were downregulated in OC cells and lower levels of Lamin A/C exhibited shorter OS **(**Figure 4D–F **)**. Next, we explored whether the deregulation of TPX2 reflected an alteration of Lamin A/C level in OC, and found that TPX2 KD significantly elevated the Lamin A/C level, whereas TPX2 OE decreased the Lamin A/C level **(**Figure 4G,H **)**. These results show that TPX2 appears to interact with Lamin A/C and regulate levels of Lamin A/C.

Previous studies have shown that elevated phosphorylation level is a pathological hallmark of Lamin degradation in cancer cells; hence, we compared the levels of p‐Lamin A/C (Ser22) in TPX2 KD and control cells, revealing that TPX2 KD decreased the level of p‐Lamin A/C (Ser 22) in OC cells **(**Figure 4I **)**.

### 3.5 Lamin A/C inhibition partially rescues apoptosis and ROS level in TPX2‐KD OC cells

**FIGURE 5:**
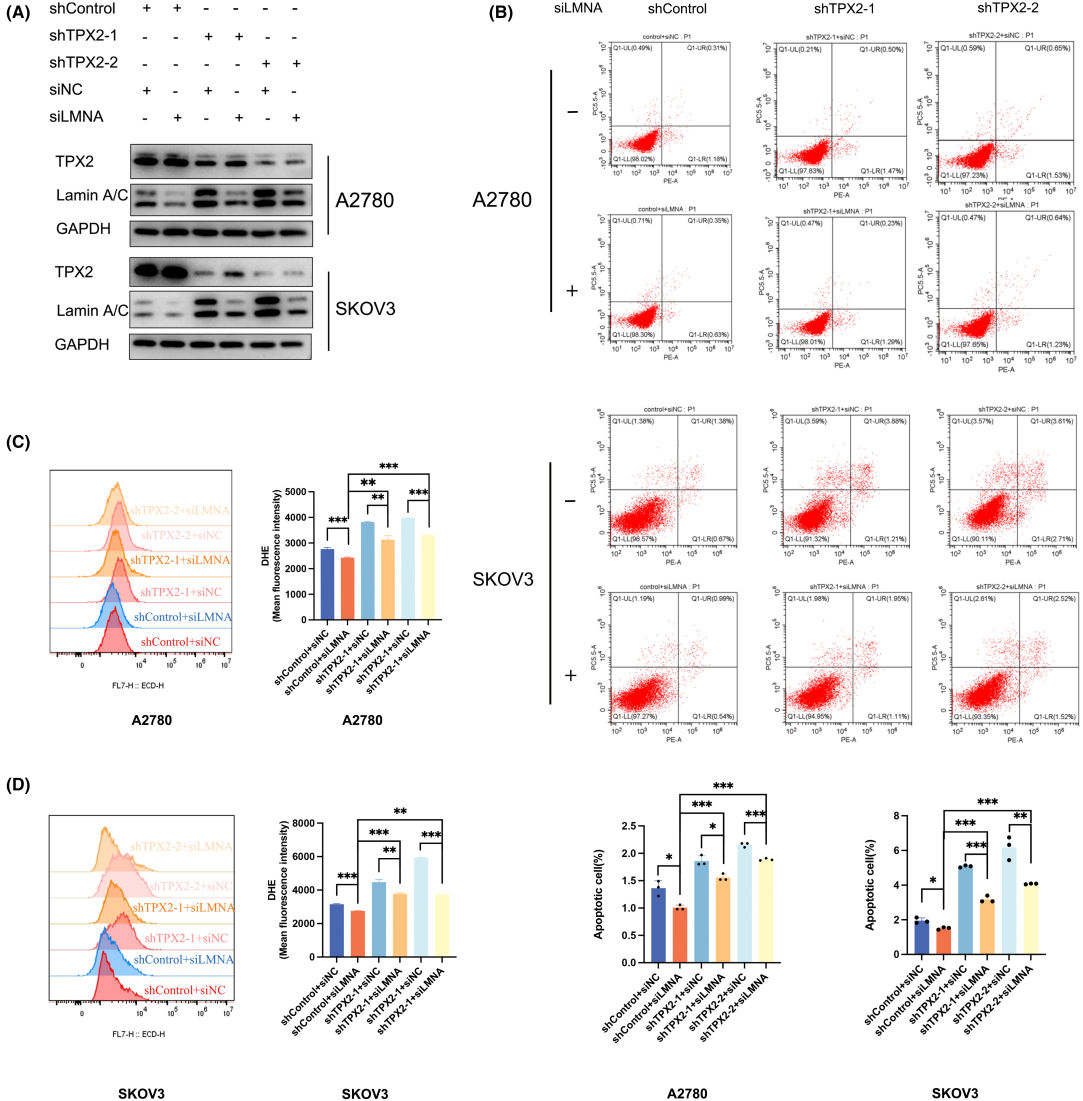
Lamin A/C inhibition partially rescues apoptosis and ROS level in TPX2‐downregulated OC cells. (A) TPX2‐depleted A2780 and SKOV3 cells were transfected with siLMNA or its negative control for 48 h. Protein levels of TPX2 and Lamin A/C were evaluated by western blotting. (B) TPX2‐KD A2780 and SKOV3 cells were transfected with siLMNA or its negative control for 48 h. Cell apoptosis was measured by flow cytometry. (C–D) TPX2‐KD A2780 and SKOV3 cells were transfected with siLMNA or its negative control for 48 h. ROS levels were detected by flow cytometry. *p<0.05, **p<0.01, ***p<0.001.

To determine the role of Lamin A/C in TPX2‐mediated tumor progression, we then transfected A2780 and SKOV3 cells with siRNA targeting the LMNA gene that codes for Lamin A/C **(**Figure 5A **)**, and performed the apoptosis and ROS assays by flow cytometry, demonstrating that Lamin A/C silencing can partially rescue OC cells from ROS production and apoptosis under TPX2 KD condition **(**Figure 5B–D **)**. These data further suggest that TPX2's role in OC pathomechanism may depend on its crosstalk with Lamin A/C in cancer cells.

## 4 DISCUSSION

Here, we delineated the critical role of TPX2 in ovarian carcinogenesis and uncovered potential disease mechanisms. TPX2 was notably overexpressed and predicted a poor prognosis in OC. Furthermore, we revealed a new association of TPX2 with Lamin A/C and demonstrated that TPX2 could mediate phosphorylation of Lamin A/C at Ser22, conferring its stability.

TPX2 has always been identified as a critical factor in mitosis and spindle assembly. Recently, numerous studies have shown that TPX2 mediates DNA damage response and participates in tumorigenesis. TPX2 can promote HCC cell proliferation by facilitating G2/M phase progression.^8^ Another study has also shown that TPX2 silencing could exert antitumor effects by targeting the PI3K/AKT signaling pathway.^12^ Furthermore, TPX2 is overexpressed in primary colorectal cancer tissues.^13^, ^14^ However, the mechanistic role of TPX2 in OC remains unclear to date. We showed that abnormal OE of TPX2 could act as an oncoprotein in OC by multiple molecular biology, and bioinformatic analyses from GEO and TCGA datasets. Considering the pro‐cancer effect of TPX2, we further assessed its clinical value. Our data showed that high TPX2 expression correlated with poor outcomes in OC patients. Functionally, TPX2 KD resulted in an attenuated proliferation of OC cells, increasing the rate of apoptosis and ROS levels. Conversely, TPX2 OE promoted OC tumorigenicity in vitro. Collectively, TPX2 may be a potential driving force for OC carcinogenesis and an attractive target for future therapies.

To further elucidate the molecular mechanism of TPX2 action, MS analysis was performed to identify relevant targets. We found Lamin A/C as a potential candidate in the TPX2 interactome. Lamins are classified into two types: A‐type and B‐type Lamins.^15^ Lamin A and C, encoded by the LMNA gene, are A‐type Lamins.^16^ They are commonly researched together and known as Lamin A/C due to their extreme similarity.^17^ Irregular expression of Lamin A/C disrupts a variety of critical cellular functions, resulting in several devastating diseases.^18^ Additionally, accumulating evidence has stated that Lamin A/C is downregulated in many carcinomas, including breast cancer,^19^, ^20^, ^21^ gastric carcinoma,^22^ colorectal carcinoma,^14^, ^23^ cervical cancer,^24^ and OC.^25^ Consistent with previous studies, our results of IHC staining also indicated that Lamin A/C levels were lower in OC, and the OS curve from the Kaplan–Meier plot showed a better prognosis in the high Lamin A/C expression group. Interestingly, we found that Lamin A/C levels were upregulated in TPX2‐KD OC cells. Considering that Lamin A/C is frequently degraded by phosphorylation,^26^ we examined the p‐Lamin A/C levels in the TPX2 KD cells, revealing that the level of phospho‐Lamin A/C (Ser 22) was significantly reduced in these cells. Functionally, siRNA‐mediated silencing of LMNA expression partially reversed the enhanced apoptosis and ROS level caused by TPX2 KD in OC cells.

In summary, our work identified an important carcinogenic role of TPX2 in OC. Mechanistically, we confirmed that TPX2 interacted with Lamin A/C, modulating its stability by regulating the level of p‐LaminA/C (Ser 22) in OC cells. These results provided insight into TPX2‐associated pathomechanisms in OC pathogenesis. Furthermore, TPX2 can be a promising biomarker for OC diagnosis and prognosis.

## AUTHOR CONTRIBUTIONS

**Xin Meng:** Investigation (equal); software (equal); validation (equal); writing – original draft (equal). **Jiazhen Cao:** Investigation (equal). **Hui Zheng:** Supervision (equal). **Xiao‐Lu Ma:** Methodology (equal). **Yanchun Wang:** Methodology (equal). **Ying Tong:** Methodology (equal). **Suhong Xie:** Methodology (equal). **Renquan Lu:** Supervision (equal). **Lin Guo:** Supervision (equal).

## FUNDING INFORMATION

This study was supported by National Natural Science Foundation of China (Grant Number: NSF‐ 82072876 and 82002618).

## CONFLICT OF INTEREST STATEMENT

The authors have no competing interests.

## ETHICS APPROVAL

All procedures were conducted in compliance with the ethical standards of the Ethics Committee of Fudan University Shanghai Cancer Center (Certification number: 050432–4‐1212B).

## Supporting information


**Table S1.** Sequences of shRNA and siRNA.

## Data Availability

The data used in this study are available from the corresponding author upon reasonable request.

## References

[1] Siegel RL , Miller KD , Fuchs HE , Jemal A . Cancer statistics. CA Cancer J Clin. 2022;72(1):7‐33. 35020204 10.3322/caac.21708

[2] Lheureux S , Braunstein M , Oza AM . Epithelial ovarian cancer: evolution of management in the era of precision medicine. CA Cancer J Clin. 2019;69:280‐304. 31099893 10.3322/caac.21559

[3] Matulonis UA , Sood AK , Fallowfield L , Howitt BE , Sehouli J , Karlan BY . Ovarian cancer. Nat Rev Dis Primers. 2016;2:16061. 27558151 10.1038/nrdp.2016.61 PMC7290868

[4] Odunsi K . Immunotherapy in ovarian cancer. Ann Oncol. 2017;28:viii1‐viii7. 29232467 10.1093/annonc/mdx444 PMC5834124

[5] Wadsworth P . Tpx2. Curr Biol. 2015;25:R1156‐R1158. 26702647 10.1016/j.cub.2015.10.003 PMC5434513

[6] Tomii C , Inokuchi M , Takagi Y , et al. TPX2 expression is associated with poor survival in gastric cancer. World J Surg Oncol. 2017;15:14. 28069036 10.1186/s12957-016-1095-y PMC5223319

[7] Matson DR , Denu RA , Zasadil LM , et al. High nuclear TPX2 expression correlates with TP53 mutation and poor clinical behavior in a large breast cancer cohort, but is not an independent predictor of chromosomal instability. BMC Cancer. 2021;21:186. 33622270 10.1186/s12885-021-07893-7 PMC7901195

[8] Wang Y , Wang H , Yan Z , et al. The critical role of dysregulated Hh‐FOXM1‐TPX2 signaling in human hepatocellular carcinoma cell proliferation. Cell Commun Signal. 2020;18:116. 32723329 10.1186/s12964-020-00628-4 PMC7388463

[9] Yan L , Li Q , Yang J , Qiao B . TPX2‐p53‐GLIPR1 regulatory circuitry in cell proliferation, invasion, and tumor growth of bladder cancer. J Cell Biochem. 2018;119:1791‐1803. 28799673 10.1002/jcb.26340

[10] Miwa T , Kokuryo T , Yokoyama Y , Yamaguchi J , Nagino M . Therapeutic potential of targeting protein for Xklp2 silencing for pancreatic cancer. Cancer Med. 2015;4:1091‐1100. 25914189 10.1002/cam4.453 PMC4529347

[11] Ma S , Rong X , Gao F , Yang Y , Wei L . TPX2 promotes cell proliferation and migration via PLK1 in OC. Cancer Biomark. 2018;22:443‐451. 29865033 10.3233/CBM-171056 PMC13078459

[12] Huang DH , Jian J , Li S , Zhang Y , Liu LZ . TPX2 silencing exerts antitumor effects on hepatocellular carcinoma by regulating the PI3K/AKT signaling pathway. Int J Mol Med. 2019;44:2113‐2122. 31638175 10.3892/ijmm.2019.4371 PMC6844623

[13] Sillars‐Hardebol AH , Carvalho B , Tijssen M , et al. TPX2 and AURKA promote 20q amplicon‐driven colorectal adenoma to carcinoma progression. Gut. 2012;61:1568‐1575. 22207630 10.1136/gutjnl-2011-301153

[14] Hufton SE , Moerkerk PT , Brandwijk R , de Bruine AP , Arends JW , Hoogenboom HR . A profile of differentially expressed genes in primary colorectal cancer using suppression subtractive hybridization. FEBS Lett. 1999;463:77‐82. 10601642 10.1016/s0014-5793(99)01578-1

[15] Shimi T , Kittisopikul M , Tran J , et al. Structural organization of nuclear lamins A, C, B1, and B2 revealed by superresolution microscopy. Mol Biol Cell. 2015;26:4075‐4086. 26310440 10.1091/mbc.E15-07-0461 PMC4710238

[16] Gruenbaum Y , Medalia O . Lamins: the structure and protein complexes. Curr Opin Cell Biol. 2015;32:7‐12. 25460776 10.1016/j.ceb.2014.09.009

[17] Lin F , Worman HJ . Structural organization of the human gene encoding nuclear Lamin A and nuclear Lamin C. J Biol Chem. 1993;268:16321‐16326. 8344919

[18] Bao H , Li HP , Shi Q , et al. Lamin A/C negatively regulated by miR‐124‐3p modulates apoptosis of vascular smooth muscle cells during cyclic stretch application in rats. Acta Physiol (Oxf). 2020;228:e13374. 31495066 10.1111/apha.13374

[19] Matsumoto A , Hieda M , Yokoyama Y , et al. Global loss of a nuclear lamina component, Lamin A/C, and LINC complex components SUN1, SUN2, and nesprin‐2 in breast cancer. Cancer Med. 2015;4:1547‐1557. 26175118 10.1002/cam4.495 PMC4618625

[20] Wazir U , Ahmed MH , Bridger JM , et al. The clinicopathological significance of Lamin A/C, Lamin B1 and Lamin B receptor mRNA expression in human breast cancer. Cell Mol Biol Lett. 2013;18:595‐611. 24293108 10.2478/s11658-013-0109-9 PMC6275779

[21] Aljada A , Doria J , Saleh AM , et al. Altered Lamin A/C splice variant expression as a possible diagnostic marker in breast cancer. Cell Oncol (Dordr). 2016;39:161‐174. 26732077 10.1007/s13402-015-0265-1 PMC13001873

[22] Wu Z , Wu L , Weng D , Xu D , Geng J , Zhao F . Reduced expression of Lamin A/C correlates with poor histological differentiation and prognosis in primary gastric carcinoma. J Exp Clin Cancer Res. 2009;28:8. 19144202 10.1186/1756-9966-28-8 PMC2632624

[23] Belt EJ , Fijneman RJ , van den Berg EG , et al. Loss of Lamin a/C expression in stage II and III colon cancer is associated with disease recurrence. Eur J Cancer. 2011;47:1837‐1845. 21621406 10.1016/j.ejca.2011.04.025

[24] Capo‐chichi CD , Aguida B , Chabi NW , et al. Lamin A/C deficiency is an independent risk factor for cervical cancer. Cell Oncol (Dordr). 2016;39:59‐68. 26537870 10.1007/s13402-015-0252-6 PMC13001887

[25] Capo‐chichi CD , Cai KQ , Simpkins F , Ganjei‐Azar P , Godwin AK , Xu XX . Nuclear envelope structural defects cause chromosomal numerical instability and aneuploidy in ovarian cancer. BMC Med. 2011;9:28. 21439080 10.1186/1741-7015-9-28 PMC3072346

[26] Zhang Y , Wang J , Huang W , et al. Nuclear Nestin deficiency drives tumor senescence via Lamin A/C‐dependent nuclear deformation. Nat Commun. 2018;9:3613. 30190500 10.1038/s41467-018-05808-y PMC6127343

